# Analysis of Reproductive Traits of Broiler Rabbits Reared in Sub-temperate Climate of Kodai Hills, Tamil Nadu, India

**DOI:** 10.14202/vetworld.2015.1045-1050

**Published:** 2015-09-08

**Authors:** S. Rajapandi, N. Ramanathan, R. Pourouchottamane, A.K. Thiruvenkadan, V. Ramesh Saravana Kumar, P.K. Pankaj, A.S. Rajendiran

**Affiliations:** 1 Department of Agriculture and Animal Husbandry, Gandhi Gram Rural Institute, Gandhigram, Dindigul, Tamil Nadu, India; 2 Southern Regional Research Centre, ICAR – CSWRI, Mannavanur, Kodaikanal, Tamil Nadu, India; 3 Department of Bio Statistics, Veterinary College & Research Institute (TANUVAS), Namakkal, Tamil Nadu, India; 4 Department of Livestock Production Management, Veterinary College & Research Institute (TANUVAS), Namakkal, Tamil Nadu, India; 5 Section of Transfer of Technology, ICAR – Central Research Institute for Dryland Agriculture,Saidabad, Hyderabad,Telangana, India

**Keywords:** litter traits, parity, rabbit, reproductive performance, season, sub-temperate

## Abstract

**Aim:**

The present study was carried out at Institute Rabbit Farm of Southern Regional Research Centre, Mannavanur, Kodaikanal, Tamil Nadu, India having sub-temperate climate with winter temperature during night hours going below 0°C with an objective of finding the influence of different factors such as breed, year, season and parity on different reproductive traits of broiler rabbits in order to come out with the best strategies for improving the productivity.

**Materials and Methods:**

A total of 1793 records (946 White Giant and 847 Soviet Chinchilla) for weight at mating (WM), weight at kindling (WK), gestation length (GL), litter size at birth (LSB) and litter size at weaning (LSW), litter weight at birth (LWB), and litter weight at weaning (LWW) were collected in the period between 2000 and 2009 and the data was analyzed using general linear model option of SAS 9.2.

**Results:**

The overall mean GL, WM, WK, LSB, LSW, LWB, and LWW were 31.68±0.04 days, 3.65±0.01 kg, 3.84±0.01 kg, 6.91±0.08, 5.49±0.09, 387.62±4.07 g, and 4.66±0.07 kg, respectively. The breed has significantly influenced GL, WK, LSW, LWB, and LWW. The LSB, LSW, LWB, and LWW were 7.05±0.11, 5.76±0.13, 399.55±5.88 g, and 4.87±0.10 kg, respectively in White Giant and corresponding values for Soviet Chinchilla were 6.78±0.11, 5.22±0.12, 375.91±5.64 g, and 4.46±0.09 kg, respectively. The year of kindling had significantly affected all the reproductive traits under study and is varying over different years. The parity significantly influenced the WM, WK, and LWW. The LWW increased from first (4.16±0.21 kg) to second parity (4.86±0.19 kg) and remained in the same range from third parity onward. WM was significantly higher in spring season (3.72±0.02) than the animals in rainy (3.59±0.02) and winter season (3.65±0.02). Better reproductive performance in terms of higher LSB, LSW, LWB, and LWW as observed in the present study might be due to conducive environmental conditions prevailing in the region.

**Conclusion:**

The significant effects of the non-genetic factors like year of kindling on all reproductive traits, season, and parity on some of the traits in rabbit breeds are indications that any future production enhancement strategy must take into consideration the environment by providing additional care, feed supplementation and better shelter management to the rabbits, so that the full genetic potential can be realized.

## Introduction

Rabbit rearing has gained momentum in the recent past, owing to their small body size, rapid growth, high prolificacy, early maturity, shorter generation interval and ability to utilize forage, and fibrous agricultural by-products [1]. Apart from its high prolificacy, the rabbit has several advantages over many other farm species, including meat being highly digestible, wholesome, tasty, low in cholesterol, sodium and, fat with high protein content [2].

The world rabbit meat production is estimated to be between 1.2 and 1.8 million tonnes and worlds’ major producer according to FAO in 2007 are China (0.6 million tonnes), Italy (0.23 million tonnes), Spain and Egypt (each 0.07 million tonnes), and France (0.055 million tonnes). In India, there has been a rising awareness in recent years on the virtues of broiler rabbit production as an alternative means of alleviating food shortages. The total rabbit population in India increased from 424 thousand in 2007 to 591.6 thousand in 2012 which is around 39.55% increase in last 5 years [3].

The production efficiency of commercial rabbit farms is largely dependent on the litter size at kindling and the survivability of the kits up to weaning [4]. The reproductive performance of rabbits is an important aspect in determining the profitability of commercial rabbit breeding. Factors such as breed, season, age, and weight of females influence the reproductive performance of animals [5].

The applied and basic research conducted in developed countries has yielded numerous reports, however, details about performance of broiler rabbits raised in India is very limited that too mainly focusing on performance under tropical climatic condition [1,6,7]; the reports on performance of rabbits in sub-temperate climate conditions of India are scanty [8]. In the present study, efforts have been made to find the influence of different factors such as breed, year, season, and parity on different reproductive traits of rabbits reared in sub-temperate climatic conditions of Kodai Hills, Tamil Nadu.

## Materials and Methods

### Ethical approval

The present study was carried out after getting approval by the Research Committee and Institutional Animal Ethics Committee.

### Study area

The present study was carried out at Institute Rabbit Farm of Southern Regional Research Centre, ICAR-CSWRI, Mannavanur, Kodaikanal, Tamil Nadu, India. The rabbit farm is located at 2030 m above mean sea level and climatic conditions prevailing in the region is sub-temperate with winter temperature during night hours going below 0°C. The average annual rainfall ranges from 1200 to 1400 mm. During summer months, mean monthly minimum and maximum temperature ranges from 12°C to 28°C and in winter months, it ranges from sub 0°C to 16°C.

### Animals

The rabbits considered for the analysis belong to Soviet Chinchilla and White Giant breeds and were reared in the cage system. The cages are made of galvanized iron and rabbits were kept in colony up to 12-16 weeks of age and later on shifted to individual cages. The rabbits were provided concentrate mixture (16% crude protein and 2400 kcal metabolizable energy) at the rate of 75 g/day up to 6^th^ week of age (weaners) and 100 g/day from 7^th^ to 12^th^ week of age (growers) and 150 g/day to the adult males in two divided doses in morning and evening. For the lactating does and kits, a concentrate mixture of 200-250 g/day was given, according to their body condition and litter size. In addition, rabbits were fed with green fodder (lucerne, oats, and grasses) depending on their seasonal availability in the afternoon at the rate of 250-300 g/animal. They were provided with clean lukewarm water *ad-libitum*. Kits and does were housed together and are weaned at 42 days of age. A standard prophylactic endo- and ecto-parasitic control schedule was applied. The females were first mated at 7-8 months of age and a day after each weaning (43^rd^ day after parturition) thereafter. Bucks were assigned to females for natural service. Care has been taken that buck and does are not having a common ancestor for last three generations in order to reduce inbreeding.

### Data collection

The data on various productive and reproductive traits generated and maintained at rabbit farm were collected for the present work. A total of 1793 records (946 White Giant and 847 Soviet Chinchilla) for weight at mating (WM), weight at kindling (WK), gestation length (GL), litter size at birth (LSB) and litter size at weaning (LSW), litter weight at birth (LWB), and litter weight at weaning (LWW)were collected in the period between 2000 and 2009. Each year was divided into three seasons, i.e. spring (March-June); rainy (July-October) and winter (November-February) seasons with average long-term temperature humidity index (THI) of 60, 61, and 56, respectively.

### Statistical analysis

All traits were analyzed using the following model:

Y_ijklm_=μ+B_i_+Y_j_+S_k_+P_l_+e_ijklm_, where Y_ijklm_ was the observed trait, μ was the population mean, B_i_ was the effect of breed (with two levels: Soviet Chinchilla and White Giant breeds), Y_j_ was the effect of year (with five levels: 2000-01, 2002-03, 2004-05, 2006-07 and 2008-09), S_k_ was the effect of season (with three levels: Spring, rainy and winter), P_l_ was the effect of parity of doe (with eight levels: From 1 to 7 parities and 8 or more parities) and e_ijklm_ was random error.

All the interactions were found to be non-significant and hence all interactions were ignored. All analyses were performed using the generalized linear model procedure of SAS9.2. Comparison of the means of the different subgroups was performed by Duncan’s multiple range tests [9].

## Results and Discussions

**Figure-1:**
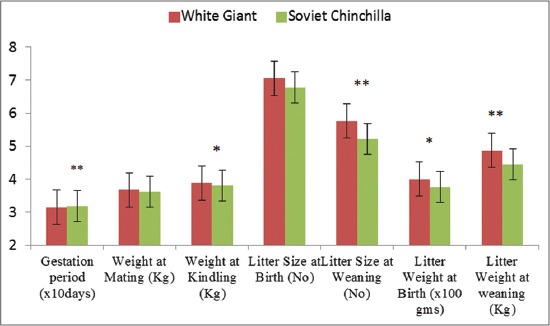
Reproductive traits in two breeds of broiler rabbits. *Significant at 5% level of significance, ** Significant at 1% level of significance.

**Figure-2:**
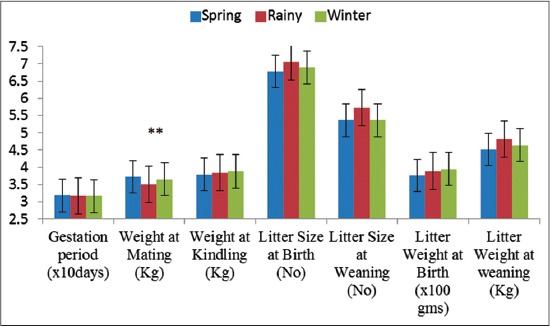
Reproductive traits in broiler rabbits under different seasons.**Significant at 1% level of significance.

The least square mean and SE of different reproductive traits like WM and WK, GL, LSB and LSW, LWB, and LWW are given in Figures-1 and 2 and Tables-1 and 2. The overall mean GL, WM, WK, LSB, LSW, LWB, and LWW were 31.682±0.04 days, 3.650±0.01 kg, 3.843±0.01 kg, 6.913±0.08, 5.485±0.09, 387.62±4.07 g, and 4.660±0.07 kg, respectively in the broiler rabbits reared under sub-temperate climatic conditions. Lower LSB, LSW, LWB, and LWW in Soviet Chinchilla (5.1±0.1, 3.7±0.1, 256.4±6.9 g, and 2465.4±73.6 g, respectively) and White Giant rabbit (5.1±0.1, 4.1±0.1, 259.6±6.1 g, and 2432.6±68.7 g, respectively) reared under tropical climatic conditions of Tamil Nadu was earlier reported [6]. The better reproductive performance obtained in the present study shows that the sub-temperate climate prevailing in the region is conducive for the rabbit rearing and the animals are optimally utilizing its genetic potential. Moreover, the rabbits in the present study were selectively bred over last two decades for better productive and reproductive traits which have resulted in better performance in the animals. Slightly lower LSB, LSW, LWB, and LWW in White Giant (5.31±0.21, 4.72±0.22, 297.33±14.41, and 3.79±0.18) and Soviet Chinchilla (5.48±0.30, 5.27±0.31, 303.22±20.9, and 4.24±0.23) was also reported in animals reared under sub-temperate climatic conditions of India [8].

**Table-1:** Reproductive traits (mean±SE) as influenced by year of birth in broiler rabbits.

Particulars	2000-01	2002-03	2004-05	2006-07	2008-09
Number	261	258	538	341	395
Gestation period (days)	32.09±0.09^a^	31.94±0.079^b^	31.48±0.06^c^	31.50±0.09^d^	31.41±0.09^e^
WM (kg)	3.44±0.03^A^	3.70±0.02^B^	3.78±0.02^C^	3.55±0.03^B^	3.76±0.03^C^
WK (kg)	3.77±0.03^A^	3.85±0.03^AB^	3.97±0.02^C^	3.75±0.04^AB^	3.86±0.04^B^
LSB (No)	6.62±0.19^A^	6.62±0.17^A^	7.48±0.13^C^	7.03±0.19^BC^	6.79±0.19^AB^
LSW (No)	5.15±0.21^AB^	4.87±0.19^A^	5.70±0.15^BC^	5.90±0.22^C^	5.81±0.22^BC^
LWB (g)	384.82±9.81^B^	404.87±8.98^C^	446.04±6.78^D^	359.46±9.89^B^	338.08±9.96^A^
LWW (kg)	3.80±0.17^A^	4.23±0.16^A^	5.22±0.12^B^	4.81±0.17^B^	5.20±0.18^B^

abcd Significant at 5% level of significance

ABCD Significant at 1% level of significance, WM=Weight at mating, WK=Weight at kindling, LSB=Litter size at birth, LWB=Litter weight at birth, LSW=Litter size at weaning, LWW=Litter weight at weaning, SE=Standard error

**Table-2:** Reproductive traits (mean±SE) in different parity for broiler rabbits.

Particulars	First	Second	Third	Fourth	Fifth	Sixth	Seventh	Eighth and above
Number	201	177	170	161	150	141	136	657
Gestation period	31.65±0.10	31.86±0.09	31.85±0.11	31.70±0.10	31.61±0.10	31.46±0.12	31.58±0.10	31.75±0.06
WM (kg)	3.30±0.04^A^	3.53±0.03^B^	3.61±0.04^BC^	3.70±0.04^C^	3.82±0.04^D^	3.72±0.04^D^	3.73±0.04^D^	3.81±0.02^D^
WK (kg)	3.55±0.04^A^	3.74±0.04^AB^	3.85±0.05^BC^	3.84±0.04^CD^	3.95±0.04^DE^	3.96±0.05^E^	3.90±0.04^E^	4.01±0.02^E^
LSB (no)	6.56±0.22	6.99±0.21	6.93±0.24	6.69±0.22	7.11±0.22	7.03±0.25	7.02±0.23	7.01±0.13
LSW (no)	5.32±0.25	5.75±0.24	5.68±0.28	5.51±0.25	5.71±0.25	5.60±0.29	5.34±0.27	5.01±0.15
LWB (g)	363.26±11.6	385.77±11.03	391.25±12.80	377.06±11.60	402.85±11.37	396.65±13.21	396.20±12.25	389.56±6.87
LWW (kg)	4.16±0.21^a^	4.86±0.19^ab^	4.83±0.23^b^	4.80±0.20^b^	5.03±0.20^b^	4.72±0.23^ab^	4.65±0.22^ab^	4.29±0.12^ab^

ab Significant at 5% level of significance

ABCD Significant at 1% level of significance, WM=Weight at mating, WK=Weight at kindling, LSB=Litter size at birth, LWB=Litter weight at birth, LSW=Litter size at weaning, LWW=Litter weight at weaning, SE=Standard error

### LSB and LSW

Litter size in rabbits is regarded as one of the most important economic traits in any breed development and improvement programs for intensive meat production. The breed significantly (p<0.05) influenced the LSW. The LSB and LSW were 7.051±0.11 and 5.759±0.13 in White Giant and corresponding values for Soviet Chinchilla were 6.778±0.11 and 5.215±0.12, respectively (Figure-1). Breed has a significant effect on number of kits weaned [6,10]. However, reports are available that LSB and LSW were not affected by breed and the values for different breeds were comparable[1,7]. A significantly lower LSW was reported in Soviet Chinchilla than in New Zealand White females (3.7 vs. 4.1 kits). Among the four breeds of rabbits reared in sub-temperate climate of Himachal Pradesh, India, the LSB and LSW were higher (p<0.05) in Grey Giant (6.3±0.3 and 6.11±0.31) followed by White giant (5.48±0.3 and 5.27±0.31), Soviet Chinchilla (5.31±0.21 and 4.72±0.33) and New Zeeland White (5.28±0.32 and 5.03±0.33) [8]. A lower LSB (4.38±0.16) and LSW (1.67±0.13)was reported in rabbits reared in hot humid tropics of India [10]. Better LSB and LSW obtained in the present study may be due to stringent selection followed over the years and better management of breeding stock. A significant effect of breed on LSB and LSW comparing Semi-giant, Chinchilla, New Zealand, and California broiler rabbit breeds was also reported [11].

LSW is an indication of the mothering ability of the doe. The higher the number of kits that survive to weaning, the better is the mothering ability of the does as the kits were solely depend on the doe for their nutrient requirements. Most maternal lines are selected based on LSW, since this trait reflects both the prolificacy and mothering ability of the doe [12].

Year of kindling was found to significantly (p<0.01) influence LSB and LSW. LSB ranged from 6.619±0.17 in 2002-03 to 7.478±0.13 in 2004-05 and LSW ranged from 4.873±0.19 in 2002-03 to 5.902±0.22 in 2006-07. This agrees with the reports [6,13] on similar rabbit breeds. It was also observed that the LSB and LSW were significantly varying over different years in Carmagnola Grey Rabbit reared in sub-temperate climate of Italy [5]. It is reported that LSW was significantly influenced by year of kindling while LSB was not affected by year effect [14]. In contrast, year has no significant influence on either LSB or LSW [7]. The variations in reproductive performance of rabbits in different years might be probably due to the differences in fodder availability and variations in management aspects followed during different years. There was, however, no clear trend with respect to the year of kindling on the parameters measured.

The season of birth had no significant effect on LSB and LSW; however, LSB and LSW were numerically higher in the litter born during rainy season (7.065±0.13 and 5.724±0.15) as compared to spring and winter. It was also observed that season has no effect on LSB or LSW [7,15]. Reports are available that season had no significant effect on LSB (6.35±0.62) or at LSW (5.39±0.58) on the rabbits reared in sub-tropical climate [1]. However,a non-significant effect of season on LSB for foreign rabbit breeds (Soviet Chinchilla, White Giant and New Zealand White) kept in the high altitude conditions of Tamil Nadu while LSW is significantly differing in various seasons have also been observed earlier [13]. Similarly, LSW was affected by season of birth [13].

The parity of doe is the number of times a doe has kindled. In the present study, parity did not have a significant effect on LSB and LSW. However, LSB and LSW were numerically lower in first parity while the values in second and subsequent parities were comparable. Litter size increases by 10-20% from the first to the second litter and then again, but by less, from the second to the third, with no change from the third to the fourth and after the fourth, the size may decrease [16].Contrary reports of LSB and LSW did not differing due to parity of the animal is also there [6,8]. In Contrast, records of parity to be significantly influencing the LSB and LSW are there [14,17].

### LWB and LWW

The breed had a significant influence over the LWB (p<0.05) and LWW (p<0.01) (Figure-1). The LWB and LWW were 399.553±5.88 g and 4.867±0.10 kg in White Giant and 375.910±5.64 g and 4.456±0.09 kg, respectively in Soviet Chinchilla. In other report, breed effect was significant among the three breeds *viz*. White giant, Soviet Chinchilla and New Zeeland White for birth weight, as well as weight at weaning [7]. A lower LWB and LWW in Soviet Chinchilla (256.4±6.9 g and 2465.4±73.6 g) and White Giant rabbit (259.6±6.1 g and 2432.6±68.7 g), respectively was also reported in animals reared under tropical climatic conditions of Tamil Nadu [6]. Slightly lower LWB and LWW in White Giant (297.33±14.41 g and 3.79±0.18 kg) and Soviet Chinchilla (303.22±20.9 g and 4.24±0.23 kg) was also observed in rabbits reared under sub-temperate climatic conditions of India [8]. Better litter weight as observed in present study might be due to conducive environmental conditions prevailing the region and THI prevailing in the region during majority of the period (except December to February) falls under thermoneutral zone of the species and also due to availability of good quality lush pasture/fodder. The thermoneutral zone for rabbit is between 15°Cand 25°C and rabbits are much more tolerant to lower temperature than higher temperature and perform optimum in this range of temperature [18].

The year had significant (p<0.01) influence on both LWB and LWW. The LWW varied from 3.801±0.17 kg in 2000-01 to 5.219±0.12 kg in 2004-05. The year effect was significantly influencing both the traits *viz*., birth weight and weaning weight of individuals which can be attributed to the factor that the availability of good quality roughage feed during the year [7]. A significant effect of year of birth on LWB and LWW is also reported earlier [6,19]. The season of kindling did not havesignificant role in affecting different litter traits and the values were comparable. In contrast, LWB and LWW were influenced by season of kindling [6,19]. They inferred that the lower LWB during summer season could be due to the limited availability of good quality green forage to the females. LWB is not influenced by season while LWW is differing significantly over various seasons [8].

In the present study, parity had significant influence on LWW. Though LWB did not vary significantly in different parity, both LWW and LWB increased from first parity (363.258±11.6 g and 4.161±0.21 kg) to second parity (385.769±11.03 g and 4.864±0.19 kg). Parity had a significant effect (p<0.01) on LWW in white giant and soviet chinchilla breeds and it increased with parity order [6]. This is in agreement with increase in milk production as parity order advanced [20]. The lower LWB and LWW in first parity animals as observed in the present study is due to the fact that earlier-parity animals continue to grow until reaching adult size and compete with the foetuses for available nutrients during pregnancy. Again, increased birth weight with increased parity is an indication of older dams’ ability to utilize feed more efficiently to support foetal development than younger ones as reported earlier [21] in cows. Higher LWB and LSW were reported for White Giant rabbits at different parities [22] reared under sub-temperate conditions of India. A significant effect of parity on LWB, as well as LWWwas observed [14,17], while parity had significantly influenced the LWB which was higher in the third parity than in the first and the second parity [23]. Litter traits were not significantly influenced by parity [8]; however, litter size and weight at birth as well as LSWin the present experiment increased with advancing parity.

### WM and WK

In the present study, both WM and WK were significantly (p<0.01) influenced by effects of year and parity (Figures-1 and 2; Tables-1 and 2). WK was significantly (p<0.05) affected by breed. WM and WK were 3.680±0.02 and 3.886±0.02 kg in White Giant and 3.621±0.01 and 3.801±0.02 kg in Soviet Chinchilla, respectively. Prolificacy (number of young per kindling) varies significantly according to several factors which may be inherent in the animal. It depends on the season and the reproductive rate imposed on the doe. In healthy does receiving normal feed and 12-14 h of light, prolificacy seems to be linked to adult size [16], hence WM plays a role in determining the litter traits. WM and WK was significantly lower in first (3.301±0.04 and 3.553±0.04 kg) and second parity (3.533±0.03 and 3.738±0.04 kg) as compared to the rabbits in other parities. WM was significantly higher in spring season (3.719±0.02) than the animals in rainy (3.591±0.02) and winter season (3.647±0.02). Reports for WM significantly different in various seasons [8] are agreeable with present findings.

### GL

The GL was significantly affected by breed (p<0.01) and year of kindling (p<0.05), while season and parity had no effect on GL (Figures-1 and 2; Tables-1 and 2). The GL in white giant and soviet chinchilla were found to be 31.534±0.05 and 31.826±0.05, respectively. Reports of the significant effect of year on GL [24] and not influenced by either breed or season effect [1] is there which is partly agreeable with present findings that season had no effect on GL. Reports of the non-significant effect of season on GL for foreign rabbit breeds like Chinchilla, White Giant and New Zealand White kept in the high altitude conditions of Tamil Nadu is also there [13]. However, GL was significantly affected by year and season of kindling but was not influenced by the order of parity [14]. In the present study, it was noted that parity had no significant effect on GL.

## Conclusion

Better reproductive performance in terms of higher LSB, LSW, LWB, and LWW as observed in present study might be due to favorable environmental conditions prevailing in the region and THI in the region during majority of the period falls under thermoneutral zone of the species and also due to availability of good quality lush pasture/fodder. The significant effects of the non-genetic factors like year of kindling on all reproductive traits, season, and parity on some of the traits in rabbit breeds are indications that any future performance improvement strategy must take into consideration the environment by providing additional care, feed supplementation and better shelter management to the rabbits so that the full genetic potential can be realized.

## Authors’ Contributions

SR, NR, VRSK, RP, and ASR designed the work. SR conducted the study. AKT and PKP helped SR and RP for statistical analysis. SR, NR, VRSK, and RP prepared the manuscript. PKP revised the manuscript for communication to the journal. All authors read and approved the final manuscript.
